# Supplementary material for: Role of xylem in root hydraulics: Functionality and implications for drought adaptation
Source: Quant Plant Biol. 2025 Oct 13;6:e42. doi: 10.1017/qpb.2025.10026 (PMC12740494; doi:10.1017/qpb.2025.10026)
Supplement: Barry et al. supplementary material [file S263288282510026Xsup001.docx]

**Methods S1: CPlantBox modelling**

To investigate how contrasting root system topologies influence whole root system conductance (*K*_rs_) and to evaluate potential limitations imposed by axial conductance on water transport, we employed the 3D whole-plant model CPlantBox (Giraud et al., 2023). This model simulates plant development as a network of interconnected segments representing various organs (e.g., leaves, pseudo-stems, crown and seminal roots), which grow by elongation or the initiation of new branches. At each time step, water flow from the soil–root interface to the plant collar, as well as the resulting *K*_rs_, is computed dynamically using an analytical solution describing flow through infinitesimal root subsegments (Meunier et al., 2017), as implemented in CPlantBox (Giraud et al., 2023; Bauer et al., 2024).

Simulations were conducted for a dicot (soybean) and a monocot (wheat) species. The species were selected based on plant-functional diversity and the availability of root architectural data. The XML-input parameter files defining the root architecture dynamics for each species were obtained from the literature (Moraes et al., 2020; Morandage et al., 2021). Whole root system conductance was dynamically simulated for both species over a 70-day period, similarly to the approach used in Baca Cabrera et al. (2024). For both species, segment-scale root hydraulic properties—radial conductivity (*k*_r_) and axial conductance (*k*_x_)—were parametrized using values from published studies and a root hydraulic property database (Doussan et al., 1998; Baca Cabrera et al., 2024). These parameters were age-dependent and varied between axial and lateral roots (Table S1).

To assess the sensitivity of *K*_rs_ to variation in segment-scale hydraulic properties, *k*_r_ and *k*_x_ were systematically modified by one order of magnitude relative to the default parametrization. This range is consistent with the spatial and temporal variability reported in the literature (Baca Cabrera et al., 2024). The resulting simulations allowed us to quantify the contribution of each property to *K*_rs_ and to identify species-specific differences in how changes in radial versus axial conductance affect water transport at the whole-root system scale.

**Table S1: *k*_r_ and *k*_x_ default parametrization**

| **Root type** | **Age (days)** | ***k*_r_ (m MPa^-1^ s^-1^)** | ***k*_x_ (m^4^ MPa^-1^ s^-1^)** |
| --- | --- | --- | --- |
| Axial roots | <10 | 2.6∙10^-7^ | 3.2∙10^-12^ |
|  | <45 | 9.4∙10^-8^ | 3.9∙10^-11^ |
|  | <100 | 2.2∙10^-8^ | 5.0∙10^-10^ |
| Lateral roots | <5 | 2.1∙10^-7^ | 1.2∙10^-12^ |
|  | <15 | 2.1∙10^-7^ | 7.1∙10^-12^ |
|  | <25 | 2.0∙10^-8^ | 2.0∙10^-11^ |

**References**

Baca Cabrera JC, Vanderborght J, Couvreur V, Behrend D, Gaiser T, Nguyen TH, Lobet G (2024). Root hydraulic properties: an exploration of their variability across scales. *Plant Direct* 8: e582

Bauer FM, Baker DN, Giraud M, Baca Cabrera JC, Vanderborght J, Lobet G, Schnepf A (2024). Root system architecture reorganization under decreasing soil phosphorus lowers root system conductance of *Zea mays*. *Annals of Botany* mcae198

Doussan, C., Vercambre, G., & Pagè, L. (1998). Modelling of the hydraulic architecture of root systems: An integrated approach to water absorption distribution of axial and radial conductances in maize. *Annals of Botany* 81, 225–232.

Giraud M, Gall SL, Harings M, Javaux M, Leitner D, Meunier F, Rothfuss Y, van Dusschoten D, Vanderborght J, Vereecken H, et al (2023). CPlantBox: a fully coupled modelling platform for the water and carbon fluxes in the soil–plant–atmosphere continuum. *in Silico Plants* 5: diad009

Meunier, F., Couvreur, V., Draye, X., Vanderborght, J., & Javaux, M. (2017). Towards quantitative root hydraulic phenotyping: Novel mathematical functions to calculate plant-scale hydraulic parameters from root system functional and structural traits*. Journal of Mathematical Biology*, 75, 1133–1170.

Moraes MT de, Debiasi H, Franchini JC, Mastroberti AA, Levien R, Leitner D, Schnepf A. 2020. Soil compaction impacts soybean root growth in an Oxisol from subtropical Brazil. *Soil and Tillage Research* 200: 104611.

Morandage S, Vanderborght J, Zörner M, Cai G, Leitner D, Vereecken H, Schnepf A. 2021. Root architecture development in stony soils. *Vadose Zone Journal* 20: e20133.
